# Resilience, Mentalizing and Burnout Syndrome among Healthcare Workers during the COVID-19 Pandemic in Serbia

**DOI:** 10.3390/ijerph19116577

**Published:** 2022-05-28

**Authors:** Teodora Safiye, Branimir Vukčević, Medo Gutić, Ardea Milidrag, Draško Dubljanin, Jakša Dubljanin, Branimir Radmanović

**Affiliations:** 1 Faculty of Medical Sciences, University of Kragujevac, Svetozara Markovića 69, 34000 Kragujevac, Serbia; m.gutic1992@gmail.com (M.G.); ardeamilidrag1@gmail.com (A.M.); draskodubljanin@gmail.com (D.D.); drjale81@gmail.com (J.D.); biokg2005@yahoo.com (B.R.); 2 High School of Culinary Arts and Tourism with Dormitory, Heroja Čajke 18, 36210 Vrnjacka Banja, Serbia; planovi@uts.edu.rs; 3 Public Health Institution Health Center “Dr Branko Zogovic”, Hridska bb, 84325 Plav, Montenegro; 4 Faculty of Medicine, University of Belgrade, Dr Subotića 8, 11000 Belgrade, Serbia; 5 Department of Pulmonology, University Clinical Hospital Center Zvezdara, Dimitrija Tucovića 161, 11120 Belgrade, Serbia; 6 Department of Cardiology, University Clinical Hospital Center Zemun, Vukova 9, 11080 Belgrade, Serbia; 7 Psychiatry Clinic, University Clinical Center Kragujevac, Zmaj Jovina 30, 34000 Kragujevac, Serbia

**Keywords:** burnout syndrome, mental health, COVID-19 frontline, mentalizing, doctors, nurses, emotional exhaustion, hypomentalizing, hypermentalizing, Serbia

## Abstract

The aim of this study was to examine whether the capacity for mentalizing and resilience among healthcare workers (HCWs) explains the degree of burnout syndrome during the COVID-19 pandemic in Serbia. The research was conducted on a sample of 406 healthcare workers (141 doctors and 265 nurses), aged 19 to 65 years (M = 40.11, SD = 9.41)—203 worked on the COVID-19 frontline, and 203 in regular clinical conditions. The Maslach Burnout Inventory was used to measure the burnout syndrome. Capacity for mentalizing was examined using the Reflective Functioning Questionnaire. The Brief Resilience Scale was used to measure resilience. The results indicated that there were negative correlations between resilience and the dimensions of burnout—emotional exhaustion (r = −0.38; *p* < 0.01) and depersonalization (r = −0.11; *p* < 0.05), and a positive correlation between resilience and personal accomplishment (r = 0.27; *p* < 0.01), as was expected. The analyses of hierarchical linear regression showed that hypomentalizing was a significant positive predictor of emotional exhaustion (ß = 0.12; *p* < 005) and depersonalization (ß = 0.15; *p* < 0.05), resilience was a significant negative predictor of emotional exhaustion (ß = −0.28, *p* < 0.01) and positive predictor of personal accomplishment (ß = 0.20; *p* < 0.01), and that the degree of explained variance of burnout dimensions was higher when resilience and hypomentalizing were included in regression models, in addition to sociodemographic variables. The findings suggest that being a woman and working on the COVID-19 frontline implies a higher burnout, while the level of burnout decreases with better socioeconomic status and more children. Resilience, capacity for mentalizing, and burnout syndrome among HCWs are interrelated phenomena, which have important professional implications.

## 1. Introduction

SARS coronavirus 2 (SARS-CoV-2) appeared in December 2019 in Wuhan, China, and quickly spread to other parts of the world, causing the COVID-19 pandemic [1], and had a major negative impact on health systems in most countries, especially on mental health and well-being of healthcare workers (HCWs), who have significant responsibility in the fight against the COVID-19 pandemic. In the midst of the COVID-19 pandemic, healthcare workers in all countries of the world, including Serbia, faced life-threatening situations, dealt with an increased workload and protection measures, cared for their own and patients’ health, followed strict protocols in the treatment of COVID patients, and had to reorganize previous work models and the implementation prevention measure [2,3]. However, the growing number of infected patients has made them very vulnerable to physical, mental, and emotional exhaustion [2]. One of the phenomena that occur in situations of increased mental and emotional exhaustion at work is burnout syndrome, so it is expected to be more frequent among HCWs in a situation of increased stress due to COVID-19 [3]. In particular, understanding the role of personality strengths, such as resilience and capacity for mentalizing, could also be very important in explaining burnout syndrome.

### 1.1. Burnout Syndrome

Systematic review and meta-analysis, which aimed to present a comprehensive picture of the prevalence of burnout syndrome and its dimensions among healthcare workers during the COVID-19 pandemic, showed that the prevalence of burnout was 52% among all healthcare workers worldwide, with nurses and/or doctors experiencing the highest levels (66%), which is higher than the rates reported in other studies performed during the past two decades (i.e., 32% to 34%) [4]. Burnout syndrome is defined as a prolonged response to chronic emotional and interpersonal stressors at work and, according to Cristina Maslach’s theory, it consists of three dimensions: emotional exhaustion, depersonalization (cynicism), and reduced personal accomplishment [5]. Emotional exhaustion is reflected in the presence of feelings of exhaustion due to work, and this is also the main, but not the only, criterion of the burnout syndrome. Depersonalization is a feature of the burnout syndrome that is characteristic of the helping professions and refers to negative responses to various aspects of work and a negative attitude towards work and colleagues, with indifference to work and alienation from the psychological state of patients. Reduced personal accomplishment is a dimension of self-assessment in the burnout syndrome, and refers to the experience of incompetence and lack of achievement and productivity at work [6]. New findings on burnout indicate that the COVID-19 pandemic most likely contributed to a significant increase in burnout levels of healthcare workers, compared to the time before the pandemic [7], as indicated by the studies conducted in France, Italy, and Spain [8].

### 1.2. Resilience

There are significant individual differences in adaptation to a stressful situation such as the COVID-19 pandemic, which depends on personality characteristics and psychological resources such as resilience. Resilience refers to an individual’s ability to return to a state of normal mental functioning after stressful or threatening events, without lasting negative consequences [9]. In a broader sense, resilience is the result of all protective factors acting to maintain an individual’s mental health in distressful circumstances that may cause severe stress or mental trauma. Protective factors can be: (1) individual factors, such as ways of coping with stress, cognitive capacity, and strength of character of the individual; (2) factors arising from the social network of the individual, such as emotional or material support provided by family or close friends; and (3) support from the wider community, such as support provided by state institutions, companies, and social organizations [7,9]. Even before the COVID-19 pandemic, high resilience was cited as a personality trait that enables healthcare professionals to easily recover from various adversities at work, which can be acquired through appropriate training programs [10,11,12]. Resilience is also cited as a personality trait that can reduce the association between burnout syndrome and mental health difficulties of healthcare professionals [13,14,15,16].

### 1.3. Capacity for Mentalizing 

Mentalizing is a form of imaginative mental activity that consists of interpreting perceived human behavior based on intentional mental states such as feelings, desires, wishes, beliefs, goals, and attitudes. Mentalizing is a process that enables individuals to correctly understand their own and other people’s behavior in interpersonal relationships, as well as to well regulate their own emotions and impulses. In direct contact with another person, the basic mental actions that an individual performs when mentalizing are making assumptions about the mental states that determine behavior, and to check them. Then the individual is aware that intentional mental states cannot be seen with the naked eye. During mentalizing, an individual has a not-knowing stance about intentional mental states and sincere curiosity that helps him/her discover them in collaboration with another person [17,18,19].

Low mentalizing capacity has been found in patients with borderline personality disorder, but other mental health disorders include difficulties in mentalizing [19,20]. In addition, in the non-clinical population, forms of impaired mentalizing capacity were examined. Two such forms were investigated in these studies: hypomentalizing and hypermentalizing. These are two qualitatively different phenomena, not extremes of the same. Hypomentalizing refers to the lack or absence of consideration of the phenomena of mental life that determine behavior, which takes place through the setting of assumptions and their verification in interpersonal interaction. Hypermentalizing refers to making too many assumptions about intentional mental states, some of which are uncritically accepted as true, even though they are not true. It is manifested as excessive certainty in the accuracy of one’s own beliefs about the nature of mental states that underlie one’s behavior [18,21]. 

### 1.4. The Theoretical Framework, Aim, and Research Hypotheses

There are findings that indicate that the capacity for mentalizing allows a correct understanding of one’s own and others’ behavior in stressful situations, which helps in overcoming stress [19,22]. Manzano-Garcia et al. [21] have shown that the capacity for mentalizing reduces the degree of burnout in entrepreneurs in Spain by reducing emotional exhaustion and cynicism. In that study, hypomentalizing had statistically significant positive correlations with emotional exhaustion and cynicism as aspects of burnout. Hypomentalizing was a statistically significant positive predictor of emotional exhaustion and cynicism in Spanish entrepreneurs [21]. Findings show that resilience and burnout in doctors are interrelated phenomena; greater resilience means less burnout, and vice versa [7,14,15]. Resilience acts as a factor that reduces doctors’ anxiety at work, as well as their exhaustion at work [23]. In addition, findings indicate that there is a negative correlation between burnout and resilience in nurses [7,16,24]. 

Taking all of this into account, the main aim of this study was to examine whether resilience and capacity for mentalizing could explain the degree of burnout in HCWs during the COVID-19 pandemic in Serbia. The following hypotheses have been set:

(1) Significant negative correlations between resilience and emotional exhaustion and depersonalization (cynicism), and a positive correlation between resilience and personal accomplishment as dimensions of burnout, are expected;
(2) Significant positive correlations between low capacity for mentalizing and emotional exhaustion and depersonalization, and significant negative correlation between weak capacity for mentalizing and the dimension of personal accomplishment, are expected;
(3) It was expected that a low capacity for mentalizing would be a positive predictor of burnout dimensions—emotional exhaustion and depersonalization, and resilience a negative predictor of the mentioned burnout dimensions.

To the best of our knowledge, this paper is the first in the world to examine the role of mentalizing and resilience in explaining the burnout syndrome in frontline and non-frontline HCWs during the COVID-19 pandemic.

## 2. Materials and Methods

### 2.1. Study Design, Sample, and Procedures

The research was conducted as a cross-sectional study. The required sample size was calculated using Raosoft Sample Size Calculator (Available online: http://www.raosoft.com/samplesize.html, accessed on 1 May 2021). According to the assumption of a margin error of 5% and a confidence level of 95%, a sample of 377 respondents was calculated. Inclusion criteria for the study sample were the following professions: doctors/specialist doctors and medical technicians/nurses. In this study, HCWs (doctors/specialist doctors and medical technicians/nurses), who had direct contact with COVID-19 and who worked in the COVID-19 zone for at least one month, were considered frontline HCWs. In addition, in this study, HCWs (doctors/specialist doctors and medical technicians/nurses) who worked in regular clinical conditions, did not have direct contact with infected patients, and did not work in the COVID-19 zone, were classified in the group of non-frontline HCWs. Other inclusion criteria were the age from 18 to 65 and residents in Serbia (doctors/specialist doctors and medical technicians/nurses), who were actively working with patients at the University Clinical Center Kragujevac in Serbia, at the time the study was conducted.

The objectives of the research were explained to potential participants at the very beginning of the questionnaire in Serbian. Participation in the research was voluntary and with informed consent, and the respondents were guaranteed the confidentiality and anonymity of the obtained data. Inadequate answers, answers of the respondents who did not meet all the criteria for inclusion in the study, and respondents who did not answer all the questions from the questionnaire, were excluded from the statistical analysis by manual review of the gathered data. 

The research was conducted from July 2021 to February 2022, during the peaks of the COVID-19 pandemic in Serbia, when the number of infected patients was the highest since the pandemic had been declared [25]. Data were collected using paper-and-pencil administration mode, at the University Clinical Center of Kragujevac in Serbia. The University Clinical Centre of Kragujevac is one of four medical centers in Serbia and serves more than 2 million people mostly from Central and Western Serbia. It contains 37 organizational units; 15 of them are clinics, 7 centers, and 15 service units, and has a capacity of 1118 beds. The complex also houses the University of Kragujevac—Faculty of Medical Sciences in Serbia [26]. 

This research is a part of a larger self-financing project (approved by the Institutional Review Board of the University of Belgrade, Faculty of Philosophy, Department of Psychology, Serbia, No: #2021-58) that examined burnout syndrome and mental health of workers during the COVID-19 pandemic in Serbia, led by the first author of this paper. This study was approved by the Ethics Committee of the University Clinical Center Kragujevac, Serbia (approval number: 01/21/279). The procedures of this study were in accordance with the Declaration of Helsinki [27]. 

### 2.2. Measures

The Maslach Burnout Inventory—MBI-HSS [5] was used to measure the burnout syndrome. The instrument consists of 22 questions that are answered by estimating the frequency of each individual item. A seven-point Likert-type scale is used, where 0 means that the statement never happens, and 6 means that it happens every day. It consists of three scales: emotional exhaustion (EE), depersonalization (DP), and personal accomplishment (PA). This questionnaire does not have a unique score, but the results of the three mentioned scales are presented and interpreted in accordance with the achieved results. The EE score was calculated by adding scores from questions 1, 2, 3, 6, 8, 13, 14, 16, and 20, and the total result on this subscale could range from 0 to 56. The DP score was calculated by adding scores from questions 5, 10, 11, 15, and 22, and could range from 0 to 30. The PA score was calculated by adding scores from questions 4, 7, 9, 12, 17, 18, 19, and 21, and could range from 0 to 48 [5]. High score on the emotional exhaustion and depersonalization subscales is directly proportional to the degree of burnout, while the score on the personal accomplishment subscale is inversely proportional, i.e., the greater the sense of personal accomplishment, the lower the level of burnout [6]. The Cronbach’s alpha coefficients on our sample for the subscales of emotional exhaustion, depersonalization, and personal accomplishment were 0.90, 0.71, and 0.74, respectively.

As in the previously mentioned study [21], the capacity for mentalizing was examined using the hypomentalizing and hypermentalizing scales of the Reflective Functioning Questionnaire of 8 items (RFQ-8) [18]. RFQ-8 consists of two subscales: (1) a subscale of certainty in one’s own assessment of mental states (RFQ-c), and (2) a subscale of uncertainty in one’s own ability to assess one’s own and others’ mental states (RFQ-u). RFQ-8 does not include a scale with an overall score because hypomentalizing and hypermentalizing do not represent poles of the same dimension, but different forms of mentalizing difficulties, as described in the introduction section. 

The RFQ-c subscale examines the degree to which a person is confident that he or she is able to accurately assess his or her own and others’ mental states. High scores on RFQ-c represent hypermentalizing. The RFQ-u subscale measures an individual’s insecurity in their own ability to assess their own and others’ mental states. High scores represent hypomentalizing, and lower scores represent optimal mentalizing [28,29]. The answers are evaluated on a seven-point Likert scale from 1—Strongly disagree, to 7—Strongly agree. The results achieved by respondents on both subscales of RFQ-8 could range from 0 to 3. RFQ showed good reliability in previous studies, Cronbach’s alpha coefficient was 0.70 or more [21,28,29,30].

The Brief Resilience Scale (BRS) developed by Smith et al. [9] was used to measure resilience. According to the mentioned authors, the Brief Resilience Scale has very good reliability, Cronbach’s alpha coefficient was above 0.8 in previous studies. The Brief Resilience Scale is one-dimensional and consists of 6 items related to the ability to recover from stressful or threatening events. Respondents chose one answer on a five-point Likert-type scale, from 1—strongly disagree, to 5—strongly agree. The total score on this scale is the arithmetic mean of all six items.

A special questionnaire for research purposes was created to assess sociodemographic, work-related, and COVID-19-related variables. Based on previous literature on the burnout syndrome in HCWs [7,13,21,31,32,33], gender (male = 1, female = 2), age, profession (doctors = 1, nurses = 2), work environment during the COVID-19 pandemic (frontline healthcare workers = 1, non-frontline healthcare workers = 2), socioeconomic status (from 1 = very poor to 5 = excellent), marital status (married = 1, single = 2), and number of children (no children = 1, one child = 2, two or more children = 3) were included as control variables in this study. 

### 2.3. Statistical Analysis

For the purposes of describing the instruments used in the survey, mean values, standard deviations, minimum, maximum, skewness, and kurtosis were used as measures of descriptive statistics. To check the reliability of these scales, Cronbach’s alpha coefficient was used as a measure of internal consistency. Pearson’s correlation coefficients and tests of their significance were used to describe the relationships between the variables. Multiple hierarchical regression analysis was used to determine whether the dimensions of mentalizing and resilience were significant predictors of burnout, three times, for each mentioned dimension of burnout as a dependent variable separately. Statistical analysis was performed using SPSS Statistics software (IBM SPSS Statistics for Windows, Version 22.0, Armonk, NY, USA). 

## 3. Results

### 3.1. Participant Characteristics

The response rate was high (85.67%): 600 questionnaires were distributed and 514 respondents (doctors/specialist doctors and medical technicians/nurses), employed at the University Clinical Center of Kragujevac in Serbia, completed the questionnaire. 

The final sample included 406 respondents who met all the criteria for inclusion in the study. Out of the total of 406 HCWs, there were 203 frontline HCWs (64 doctors and 139 nurses) and 203 non-frontline HCWs (77 doctors and 126 nurses); 267 female and 139 male respondents participated in this study. The average age of the sample was 40.11 ± 9.41 years. There were 141 doctors and 265 nurses. The age of doctors and nurses ranged from 26 to 62 years and 19 to 61 years, respectively. Out of 406 respondents, 291 (71.7%) were married, and 115 (28.3%) were single; 120 (29.6%) respondents did not have children, 111 (27.3%) respondents had one child, and 175 (43.1%) stated that they had two or more children. On a scale from 1 to 5, the largest number of respondents, 229 (56.4%) rated their socioeconomic status as good (score 3), 78 (19.2%) as very good, 16 (3.9%) as excellent, 71 (17.5%) as poor, and 12 (3%) as very poor.

### 3.2. Measures of Descriptive Statistics of the Resilience, Mentalizing, and Burnout Dimensions

Table 1 shows measures of descriptive statistics and the scale reliability. The values of skewness and kurtosis ranged from −1 to 1, and indicated that the forms of score distributions on these scales did not deviate significantly from the shape of the normal distribution, except for the skewness of the hypomentalizing subscale (RFQ-u), which indicated that most respondents had below-average values of hypomentalizing (skew = 1.21). All instruments used in this study had satisfactory or good reliability, expressed as the α coefficient of internal consistency (Cronbach’s alpha), as was expected.

### 3.3. The Relationships among Resilience, Mentalizing, and Burnout Dimensions

The correlations between the variables are shown in Table 2. These findings suggest that with a higher degree of resilience of HCWs, their emotional exhaustion was lower (r = −0.38; *p* < 0.01), the degree of depersonalization was lower (r = −0.11; *p* < 0.05), and the experience of personal accomplishment at work was higher (r = 0.27; *p* < 0.01). With a greater hypermentalizing—as the higher degree of certainty in one’s own ability to assess intentional mental states, the experience of personal accomplishment at work was higher (r = 0.32; *p* < 0.01), the level of emotional exhaustion decreased (r = −0.21; *p* < 0.01), and the level of depersonalization also decreased (r = −0.23; *p* < 0.01). With a higher level of hypomentalizing, or in other words, with a lower capacity for mentalizing, the degree of emotional exhaustion increased (r = 0.24; *p* < 0.01); furthermore, the degree of depersonalization increased (r = 0.25; *p* < 0.01), while decreasing the experience of personal accomplishment at work (r = −0.20; *p* < 0.01).

The findings showed that some control variables were important for burnout, as obtained in previous studies [21,31,34]. The findings suggested that, with the female gender, there was more emotional exhaustion (r = 0.38; *p* < 0.01) and depersonalization (r = 0.19; *p* < 0.01), and at the same time, a lower degree of resilience (r = −0.18; *p* < 0.01). The findings showed that the more children the respondents have, the lower their depersonalization (r = −0.17; *p* < 0.01), and they had a stronger experience of personal accomplishment at work (r = 0.21; *p* < 0.01), and a higher degree certainty in their own power to assess intentional mental states, and therefore more hypermentalizing (r = 0.11; *p* < 0.01). With the experience of higher socio-economic status, the respondents experienced less emotional exhaustion (r = −0.22; *p* < 0.01), and greater personal accomplishment at work (r = 0.10; *p* < 0.05). The finding suggested that nurses had more emotional exhaustion than doctors (r = 0.14; *p* < 0.01). It was shown that emotional exhaustion decreased in non-frontline HCWs (r = −0.30; *p* < 0.01), as well as depersonalization (r = −0.25; *p* < 0.01), or in other words, in frontline HCWs both emotional exhaustion and depersonalization increased.

Table 3 shows the results of hierarchical linear regression analyses. In each analysis and for each control and predictor variable, the variance inflation factor (VIF) was less than five, indicating that there were no severe multicollinearity problems.

The value of the explained variance of the dependent variable emotional exhaustion in the model with control variables was 22%, while with control and predictor variables together, it increased to 32%, mostly due to resilience (ß = −0.28; *p* < 0.01) and hypomentalizing (ß = 0.12; *p* < 0.05). The findings indicated that resilience reduces emotional exhaustion. In addition, as an aspect of low capacity for mentalizing, hypomentalizing increased depersonalization. Gender (ß = −0.30; *p* < 0.01) and work environment (ß = −0.21; *p* < 0.01) significantly contributed to explaining the variance of emotional exhaustion. Female gender and working on the COVID-19 frontline implied greater emotional exhaustion compared to the male gender and working outside the COVID-19 frontline.

The value of the explained variance of the dependent variable depersonalization in the model with control variables was 10%, while with control and predictor variables together it increased to 14%, mostly due to hypomentalizing, which was a statistically significant predictor (ß = 0.15; *p* < 0.05). The findings suggested that as an aspect of low capacity for mentalizing, hypomentalizing increased emotional exhaustion. Gender, number of children, and work environment as control variables contributed to the explanation of the depersonalization variance of approximately 10%. The findings suggested that there was a higher degree of depersonalization among females, and that there was a higher degree of depersonalization when working on the COVID-19 frontline. In addition, the finding indicated that HCWs with more children experience a lower degree of depersonalization as a burnout dimension.

The value of the explained variance of the dependent variable personal accomplishment in the model with control variables was 5%, while with the control and predictor variables together, it increased to 16%. Statistically significant predictors were resilience (ß = 0.20; *p* < 0.01) and hypermentalizing (ß = 0.23; *p* < 0.01). The finding indicated that greater resilience implied a higher degree of personal accomplishment. The finding that the variable of hypermentalizing was an important predictor of personal accomplishment experience at work, indicated that the degree of confidence in one’s own ability to accurately assess intentional mental states and the degree of confidence in one’s own success at work, increased together, or decreased together. The finding that the number of children was a significant predictor of personal accomplishment (ß = 0.18; *p* < 0.01), suggested that, with a higher number of their children, HCWs also had a stronger experience of success and efficiency in their work.

Socioeconomic status in the model with only control variables was a significant negative predictor of emotional exhaustion (ß = −0.14; *p* < 0.01) and a positive predictor of personal accomplishment (ß = 0.13; *p* < 0.01), which means that higher socioeconomic status implied less emotional exhaustion and stronger personal accomplishment experience. With the introduction of predictor variables into the model, socioeconomic status ceased to be a significant predictor of burnout dimensions.

Profession, marital status, and age of respondents were not significant predictors of burnout dimensions.

## 4. Discussion

In this paper, the relationships between resilience, mentalizing, and burnout in HCWs were examined, with the main goal of determining whether resilience and mentalizing capacity explain the degree of burnout. The results confirmed the expectation that there are significant negative correlations between resilience and burnout dimensions—emotional exhaustion and depersonalization, and a positive correlation between resilience and personal accomplishment as burnout dimensions. These findings are consistent with the previous research, which showed that the developed resilience of HCWs, implied personal skills and other opportunities to maintain a good mood after stressful circumstances, level-headedness, and correct judgment [7,10,11,12,14,15]. The findings of this study indicate that resilience in HCWs includes knowledge and skills that protect HCWs from experiencing emotional overload and exhaustion at work, which includes different experiences of frustration and stress in working with patients. In addition, the findings from the correlation matrix indicate that the resilience of HCWs implies fewer experiences of depersonalization in which patients are cynically belittled and perceived as objects. The results of the regression analysis, which showed that resilience was a significant negative predictor of emotional exhaustion and a positive predictor of personal accomplishment, are in line with the above, because they even more strongly suggest that resilience prevents burnout.

The expectations that there are significant positive correlations between subscale RFQ-u with emotional exhaustion and depersonalization, and a significant negative correlation between hypomentalizing and personal accomplishment, have been confirmed. The hypothesis that hypomentalizing explains part of the variance of emotional exhaustion and depersonalization has been confirmed. This is in line with the above-mentioned findings of Manzano-Garcia et al. [21]. Good mentalizing capacity implies that during direct communication, empathy, active listening, and authentic curiosity about mental states are expressed, both one’s own and the interlocutor’s. Hypomentalizers, instead of revealing objective facts about the reasons for their behavior through open communication with others, usually judge intentional mental states by “guessing”, referring to general laws and their previous experience, which leads to wrong conclusions. Lack of mentalizing reduces the ability of HCWs to understand their own and others’ behavior at work, which leads to interpersonal misunderstandings, conflicts, professional frustrations, job dissatisfaction, and results in treating colleagues at work and patients in a cynical way, as if they were objects and not human beings. This is in line with previous findings that a good capacity for mentalizing is a protective factor of mental health [17,20,22]. It was not expected that there would be a negative association of hypermentalizing (subscale RFQ-c) with emotional exhaustion and depersonalization, and a positive association with personal accomplishment, nor was hypermentalizing expected to be a positive predictor of personal accomplishment experience. By the nature of their work, HCWs must express compassion and sincere interest in the patient’s condition, as well as show confidence in their observations. Thus, the scale of hypermentalizing by the respondents in this sample is understood as a scale of certainty in their own assessments of intentional mental states, and not as a scale that examines confidence in their own power to make their own assessments of mental states absolutely accurate, leading to hypermentalizing. A hypermentalizer is a person who builds extensive theories about their own and others’ mental states that are not based on testing assumptions and facts [20,21], while it is known that HCWs tend to base their assumptions on the factors they are checking and in which they must be as sure as possible.

At the end of this section, we will talk about the importance of control variables. As in the previous studies [21,34], in this study, the female gender significantly explains the degree of emotional exhaustion as a dimension of burnout. Burnout syndrome is often gender-determined in the social perception of the phenomenon due to research that most often links burnout as being a female experience. However, findings that women experience more burnouts than men may be associated with gender role stereotypes, but may also reflect gender mixing with profession (HCWs and especially medical technicians/nurses are more often female) [6]. Findings of our research indicate that with the increase in the number of children, HCWs have less depersonalization and more personal accomplishment at work, which means that more children mean less burnout among HCWs. This is in line with earlier findings stating that more children mean less burnout because the responsibility of raising children does not emphasize, but reduces emotional exhaustion and feelings of work overload often experienced by HCWs [35,36].

Burnout syndrome and mental health of HCWs fighting on the front lines of the COVID-19 pandemic are a major concern in the fields of occupational health and public health, and a problem that requires attention [37]. Studies have reported that the prevalence of burnout among HCWs has further increased in various countries during the COVID-19 pandemic [31,34,37,38], and that HCWs who engaged in the care of COVID-19 patients have had significantly higher burnout rates than those who did not [38,39]. Our findings showed that working on the COVID-19 frontline significantly explains the degree of emotional exhaustion and depersonalization, which was expected and in accordance with the findings of other research [33,39,40]. The work of HCWs on the front lines of the COVID-19 pandemic is a traumatic experience that includes perceiving one’s own vulnerability and situations in which patients cannot be helped much, which increases stress, frustration, dissatisfaction, and job exhaustion [39]. The global COVID-19 pandemic has once again placed this vulnerable worker population directly in the eye of the storm, making it vital to immediately and optimally support their well-being in order to mitigate potentially devastating mental health consequences. We hope that our findings will help in the management and planning of measures to alleviate mental health concerns among frontline HCWs in the event of future pandemic scenarios. 

### 4.1. Practical Implications

Bearing in mind that HCWs are the most important and the most burdened group in the fight against the COVID-19 pandemic, several important implications of this research should be pointed out. First, HCWs need to be provided with resilience training programs. Such programs were developed even before the pandemic [10,11,12]. Secondly, since hypomentalizing increases the level of burnout, additional research could be conducted in the future that would reveal how the capacity for mentalizing of HCWs is manifested in relationships with patients and colleagues. The idea is to incorporate such knowledge into interpersonal skill training programs at work. Such training programs should be accompanied by resources or psychological support programs for the well-being of health professionals during public health emergencies, including the COVID-19 response.

### 4.2. Limitations and Future Directions

Since this is a study of correlation design, it cannot produce knowledge about cause-and-effect relationships. Longitudinal research is needed to solve this problem. Since we used a self-reporting questionnaire in data collection, self-reporting bias may be present [1]. Another limitation that must be considered is the single-centered nature of the study, which does not allow generalization of the results so that they would be valid for the entire population of HCWs. In addition, more detailed research is needed to reveal the sources of resilience and the role of capacity for mentalizing in the context of the job of HCWs, in order to create and modify their training programs, as already mentioned. In addition to the above limitations, it should be borne in mind that this is the first research in the world that has theoretical and practical significance for understanding the relationship between mentalizing, burnout syndrome, and resilience in HCWs.

## 5. Conclusions

The key conclusions of this study are that the resilience in HCWs reduces their emotional exhaustion and increases the experience of personal accomplishment at work, thus preventing their burnout; and that hypomentalizing in HCWs increases their emotional exhaustion and depersonalization, and thus encourages the burnout.

Resilience, capacity for mentalizing, and burnout syndrome among HCWs are interrelated phenomena, which have important professional implications. With greater resilience and a good capacity for mentalizing, the level of burnout syndrome in HCWs decreases. Education and training programs for HCWs should include knowledge and skills that are important for the resilience of HCWs in a pandemic, as well as those that provide a good capacity for mentalizing.

## Figures and Tables

**Table 1:** Descriptive statistics of used measures.

Scale	Min.	Max.	Mean	SD	Skew	Kurt	*α*
Depersonalization (DP)	0	29	8.82	6.42	0.68	−0.25	0.71
Emotional exhaustion (EE)	0	54	35.68	11.83	−0.68	0.03	0.90
Personal accomplishment (PA)	8	48	37.51	6.33	−0.66	0.96	0.74
Resilience (BRS)	1.00	5.00	3.21	0.74	−0.08	0.00	0.76
Hypermentalizing (RFQ-c)	0.00	3.00	1.19	0.88	0.35	−0.96	0.82
Hypomentalizing (RFQ-u)	0.00	2.50	0.57	0.62	1.21	0.68	0.70

**Table 2:** Correlations between the investigated variables.

	EE	DP	PA	BRS	RFQ-c	RFQ-u	Gender	Age	Mar.S.	N.Chil.	SE.S.	Profession
DP	0.46 **											
PA	−0.22 **	−0.28 **										
BRS	−0.38 **	−0.11 *	0.27 **									
RFQ-c	−0.21 **	−0.23 **	0.32 **	0.36 **								
RFQ-u	0.24 **	0.25 **	−0.20 **	−0.27 **	−0.61 **							
Gender	0.38 **	0.19 **	0.02	−0.18 **	−0.11 *	0.13 **						
Age	0.02	−0.10 *	0.17 **	0.07	0.18 **	−0.15 **	0.02					
Mar.S.	−0.06	0.09	−0.07	0.08	0.01	0.05	−0.06	−0.18 **				
N.Chil.	0.00	−0.17 **	0.21 **	−0.06	0.11 *	−0.06	0.12 **	0.46 **	−0.48 **			
SE.S.	−0.22 **	−0.07	0.10 *	0.19 **	0.07	−0.13 **	−0.13 **	−0.10 *	0.01	−0.05		
Profession	0.14 **	0.06	0.04	−0.09	−0.06	0.12 *	0.47 **	−0.04	−0.07	0.17 **	−0.10 *	
W.E.	−0.30 **	−0.25 **	0.02	0.08	0.13 **	−0.11 *	−0.15 **	0.08	−0.02	0.07	0.15 **	−0.06

Note: ** *p* < 0.01, * *p* < 0.05, EE—emotional exhaustion, DP—depersonalization, PA—personal accomplishment, BRS—brief resilience scale, RFQ-c—reflective function questionnaire certain, RFQ-u—reflective function questionnaire uncertain, Mar.S.—marital status, N.Child.—number of children, SE.S.—socioeconomic status, W.E.—work environment (COVID-19 frontline or non-frontline).

**Table 3:** Hierarchical linear regression analysis of the relationship among burnout dimensions, resilience, and mentalizing.

	Outcome Variable: Emotional Exhaustion						Outcome Variable: Depersonalization						Outcome Variable: Personal Accomplishment					
	Control Variables			Control Variables and Predictors			Control Variables			Control Variables and Predictors			Control Variables			Control Variables and Predictors		
	*ß*	*t*	VIF	*ß*	*t*	VIF	*ß*	*t*	VIF	*ß*	*t*	VIF	*ß*	*t*	VIF	*ß*	*t*	VIF
Gender	**0.35** **	7.07	1.33	**0.30** **	6.38	1.36	**0.18** **	3.45	1.33	**0.16** **	3.06	1.36	0.00	0.12	1.33	0.06	1.13	1.36
Age	0.04	0.79	1.33	0.09	1.97	1.37	−0.01	−0.20	1.33	0.02	0.49	1.37	0.10	1.94	1.33	0.04	0.80	1.37
Mar.S.	−0.08	−1.60	1.31	−0.06	−1.45	1.33	0.01	0.25	1.31	0.01	0.27	1.33	0.03	0.63	1.31	0.00	0.06	1.33
N.Chil.	−0.08	−1.45	1.69	−0.10	−1.97	1.71	**−0.17** **	−2.81	1.69	**−0.16** **	−2.75	1.71	**0.18** **	2.97	1.69	**0.18** **	3.08	1.71
Profession	−0.05	−1.00	1.34	−0.05	−1.06	1.35	−0.01	−0.29	1.34	−0.02	−0.47	1.35	0.02	0.45	1.34	0.02	0.37	1.35
SE.S.	**−0.14** **	−3.28	1.05	−0.08	−1.94	1.10	−0.02	−0.54	1.05	−0.00	−0.01	1.10	**0.13** **	2.62	1.05	0.08	1.68	1.10
W.E.	**−0.23** **	−5.13	1.06	**−0.21** **	−5.17	1.06	**−0.21** **	−4.39	1.06	**−0.19** **	−4.12	1.06	−0.01	−0.26	1.06	−0.03	−0.82	1.06
BRS				**−0.28** **	−6.23	1.23				−0.01	−0.26	1.23				**0.20** **	4.08	1.23
RFQ-c				0.03	0.55	1.78				−0.07	−1.27	1.78				**0.23** **	3.90	1.78
RFQ-u				**0.12** *	2.41	1.67				**0.15** *	2.56	1.67				0.01	0.24	1.67
R^2^	**0.24**			**0.33**			**0.12**			**0.16**			**0.07**			**0.18**		
adj. R^2^	**0.22**			**0.32**			**0.10**			**0.14**			**0.05**			**0.16**		
F Ch.	**17.93** **			**19.17** **			**8.05** **			**6.72** **			**4.36** **			**18.84** **		

Note: ** *p* < 0.01, * *p* < 0.05, EE—emotional exhaustion, DP—depersonalization, PA—personal accomplishment, BRS—brief resilience scale, RFQ-c—reflective function questionnaire certain, RFQ-u—reflective function questionnaire uncertain, Mar.S.—marital status, N.Child.—number of children, SE.S.—socioeconomic status, W.E.—work environment (COVID-19 frontline or non-frontline); statistically significant correlations are bolded.

## Data Availability

The data presented in this study are available on request from the corresponding author.

## Declaration

This paper was the result of a larger self-financing project led by Teodora Safiye during Ph.D. studies, and is part of her doctoral dissertation.
